# Medulla oblongata dominated synaptic density network degeneration in amyotrophic lateral sclerosis

**DOI:** 10.1016/j.nicl.2025.103814

**Published:** 2025-06-18

**Authors:** Ting Zou, Manliu Hou, Honghao Han, Xuyang Wang, Huafu Chen, Yongxiang Tang, Rong Li, Shuo Hu

**Affiliations:** a The Clinical Hospital of Chengdu Brain Science Institute, School of Life Science and Technology, University of Electronic Science and Technology of China, Chengdu 610054, PR China; b MOE Key Laboratory for Neuroinformation, High-Field Magnetic Resonance Brain Imaging Key Laboratory of Sichuan Province, University of Electronic Science and Technology of China, Chengdu 610054, PR China; c Department of Nuclear Medicine, Xiangya Hospital, Central South University, Changsha 410008, PR China; d National Clinical Research Center for Geriatric Diseases, Xiangya Hospital, Central South University, Changsha 410008, PR China; e Department of Nuclear Medicine, Inselspital, University Hospital Bern, Bern, Switzerland; f Key Laboratory of Biological Nanotechnology of National Health Commission, Xiangya Hospital, Central South University, Changsha 410008, PR China

**Keywords:** Amyotrophic lateral sclerosis, Synaptic density similarity network, Causal synaptic covariance network, Medulla oblongata, Network degeneration

## Abstract

**Background:**

Amyotrophic lateral sclerosis (ALS) is a brain network disorder closely associated with synaptic loss in the upper and lower motor neurons. However, the *in vivo* synaptic network changes and their progressive processes remain unclear. Here, we aim to investigate the synaptic density network connectivity and the likely sequences of synaptic loss in patients with ALS.

**Methods:**

We examined data from 21 patients diagnosed with ALS and 25 sex- and age-matched healthy controls (HCs) who underwent PET imaging with the SV2A radioligand [^18^F]SynVesT-1. The individual synaptic density similarity network was constructed for each patient by calculating the similarity between interregional synaptic density distributions. The synaptic network connectivity changes were investigated, followed by an examination of the local synaptic density in regions that showed significant network alterations. Finally, we constructed the voxel-wise and ROI-wise causal synaptic covariance network (cSCN) by applying Granger causality analysis. This allowed us to identify the sequence of synaptic loss in these brain regions.

**Results:**

We observed an overall decrease in synaptic density network connectivity in ALS patients compared to controls, with the highest nodal degree in the right medulla oblongata. Specifically, the reduced connections were dominantly between the medulla oblongata and the striatum, frontal lobe, occipital lobe, as well as between the striatum and the frontal lobe, occipital lobe. Furthermore, patients with ALS displayed significantly synaptic loss in those brain regions. The cSCN analyses showed that as the disease progresses, the cortical synaptic loss sequences of ALS extend from the medulla oblongata to the regions including the striatum, frontal lobe, occipital lobe, and parietal lobe.

**Conclusions:**

These findings suggest that synaptic density network degeneration in ALS may follow a bottom-up transmission pattern, primarily involving in the medulla oblongata-striatum-neocortex network, which have the potential to capture new network-based targets for clinical therapy in the progression of ALS.

## Highlights

• ALS patients showed decreased synaptic density network connectivity.
• ALS patients displayed significantly synaptic loss in those brain regions.
• Synaptic degeneration in ALS may in medulla oblongata-striatum-neocortex network.

## 1 Introduction

Amyotrophic lateral sclerosis (ALS) is a progressive neurodegenerative disease that affects the upper and lower motor neurons in the motor cortex, the brainstem nuclei and the anterior horn of the spinal cord, leading to paralysis and eventually to death within 3 to 5 years after the symptom onset (De Lorenzo et al., 2023, Feldman et al., 2022, Roche et al., 2012). Disruptions in synapse can have severe effects on synaptic transmission, leading to altered network interactions and ultimately the clinical manifestation of disease (Henstridge et al., 2016). Despite considerable progress has been made in understanding the abnormal mechanisms of synaptic transmission between neurons in established animal models of ALS (Hoye et al., 2018, Lépine et al., 2022), little is known about the *in vivo* macroscale synaptic network changes and their progressive relationship in patients with ALS.

Human post-mortem studies using electron microscopy with high-resolution techniques have shown that the loss of synapses in ALS is not occur uniform but more apparent in vulnerable brain areas such as the prefrontal cortex, brainstem, striatum, postcentral neocortex and temporal lobe (Brettschneider et al., 2013, Henstridge et al., 2018). A novel marker, [^18^F]SynVest-1 PET, which is a SV2A radioligand has been shown to have high brain uptake, appropriate tissue kinetics, and high levels of specific binding in healthy volunteers (Naganawa et al., 2021, Tang et al., 2022b, Tang et al., 2022c). Recently, our first [^18^F]SynVesT-1 PET study of ALS extends the previous findings by tracking regional synaptic loss in the cingulate cortex, insula, and temporal gyrus (Tang et al., 2022a). Loss of synapses is likely to affect network organization through either local or long-distance connected brain regions. Although the distance between the involved regions is often considerable, the affected neurons in these regions are connected by axonal projections, and the physical contacts between nerve cells along axons are important for dissemination of ALS pathology (Braak et al., 2013). So far, brain network studies using magnetic resonance imaging (MRI) have disclosed increased functional connectivity within the frontal, temporal, parietal and subcortical regions(Geevasinga et al., 2017) and decreased structural connectivity in sensorimotor, basal ganglia, frontal, and parietal areas in patients with ALS (Basaia et al., 2020). Inspired by MRI-based network analysis, the Kullback-Leibler divergence similarity estimation (KLSE) method has recently been utilized to construct individual brain metabolic connectomes for [^18^F]SynVesT-1 PET images (Li et al., 2025). This method has the potential to accurately quantify the *in vivo* changes in synaptic networks in patients with ALS.

Although the cross-sectional cerebral neuroimaging signature of ALS is well understood as the neuro system degeneration with the aforementioned brain regions involvement, the progressive degeneration in ALS remains poorly understood. Here, we present evidence of the synaptic connectivity patterns and the potential ordering of synaptic loss in ALS. In contrast to stage-specific comparative analysis, which maps disease progression based on cross-sectional data (Shima et al., 2012), causal structural covariance network (CaSCN) analysis can help to describe potential neurodegenerative patterns at the network level (Guo et al., 2020, Li et al., 2021). Similar to the CaSCN, if cross-sectional [^18^F]SynVesT-1-PET data are associated with temporal information by sequencing the data according to disease progression information, we could construct causal synaptic covariance network (cSCN) to characterize the causal relationships of synaptic loss in these regions.

In our study, we firstly applied the KLSE algorithm to construct the individual-level synaptic connectome of [^18^F]SynVesT-1 PET images and compared the differences in synaptic network connectivity between patients and controls. Then, similar to the causal structural covariance network (CaSCN) (Guo et al., 2020, Li et al., 2021), we established voxel-wise and ROI-wise causal synaptic covariance network (cSCN) in cross-sectional [^18^F]SynVesT-1-PET data to characterize the causal relationships of synaptic loss in these regions.

## 2 Methods

### 2.1 Samples

The data of all participants with ALS and healthy controls (HCs) were obtained from the Department of Neurology and Nuclear Medicine, Xiangya Hospital, Central South University from 18 December 2019 to 21 April 2021, as described in the previous study (Tang et al., 2022a). A total of 21 right handed patients with ALS were diagnosed with clinically definite, probable, or probable laboratory-supported ALS by at least two experienced neurologists according to the revised El Escorial criteria 2015 (Ludolph et al., 2015). All 21 ALS patients were incident cases, recruited upon initial diagnosis. Each patient had been received standard-of-care riluzole and edaravone from diagnosis onward. Twenty-five sex- and age- matched healthy individuals with normal cognitive function were enrolled as HCs. All control subjects ruled out a history of neurological disease and a family history of cerebellar disorders.

### 2.2 Standard protocol approvals, registrations, and patient consents

The local ethics committee of the recruiting center approved by the ethics committee and the expert committee of Xiangya Hospital, Central South University. The number of the approval is 202106134. Written informed consent was obtained from all participants.

### 2.3 Measures

Demographic and clinical data, including age, gender, family history, years of education, age at onset, disease duration, Amyotrophic Lateral Sclerosis Functional Rating Scale-Revised (ALSFRS-R) scores, were collected by specialists. In addition, all patients with ALS were diagnosed with mild-to-severe stages of the disease (King stages 1–5) and a high score suggested advanced disease stage (Roche et al., 2012). The cognitive status was evaluated using Edinburgh Cognitive and Behavioural ALS Screen (ECAS) (Abrahams et al., 2014, Ye et al., 2016). The interval between clinical diagnosis and [^18^F]SynVesT-1 PET imaging ranged from 1 to 658 days (mean ± SD: 162.3 ± 230.5 days). The clinical characteristics for each participant are detailed in the Supplement Table 1.

## 3 Data acquisition

[^18^F]SynVesT-1 was synthesized using the methods as described in the previous studies (Li et al., 2018, Naganawa et al., 2021). For all participants, 5 min three-dimensional static PET images were obtained approximately 60 min after [^18^F]SynVesT-1 (3.7 MBq/kg) was injected intravenously through the cubital vein over 1  min. PET/computed tomography (CT) images of all ALS patients and HCs were acquired using a Discovery Elite PET/CT scanner (GE Healthcare). To minimize the potential pharmacological interference, all participants abstained from SV2A-targeting drugs for at least 24  h prior to scanning, if indicated. Additionally, participants were placed in the PET scanner so that slices were parallel to the canthomeatal line. All images were reconstructed as a 256 × 256 *trans*-axial matrix (35  cm field of view) using the 3D VUE Point (GE Healthcare) ordered-subset expectation–maximization algorithm with six iterations and six subsets, which produced 47 transaxial images at 3.25- mm intervals. The full width of the scan at half-maximum was 5.4  mm. A low-dose CT scan was obtained simultaneously for photon attenuation correction.

## 4 Data preprocessing

All processing processes was performed using Statistical Parametric Mapping software (SPM12, http://www.fil.ion.ucl.ac.uk/spm/). After visual inspection for folding and movement artifacts, the individual [^18^F]SynVesT-1 PET image volumes were spatially normalized into standard stereotactic space using an in-house [^18^F]SynVesT-1 PET template with voxel sizes of 2 mm × 2 mm × 2 mm and smoothed with a 3-dimensional Gaussian filter (FWHM 8 mm^3^). The standard uptake value (SUV) of each subject's PET image was calculated using the proportional method to prevent individual differences in brain tracer uptake from masking local changes.

## 5 Synaptic density similarity network construction

Individual synaptic density similarity network was constructed using the template defined jointly by AAL-90 and 6 brainstem regions (the bilateral medulla oblongata, midbrain, pons) derived from the 268 brain mask (https://bioimagesuiteweb.github.io/webapp/). We used Kernel Density Estimation (KDE) to estimate probability density functions for the [^18^F]SynVesT-1 PET uptake distribution in each ROI with automatically chosen bandwidths according to the studies (Mertens et al., 2022, Wang et al., 2020). This method provides a quantitative evaluation of the tracer distribution across the brain, and a high connectivity strength between nodes representing a more uniform uptake in the corresponding brain regions (Mertens et al., 2022). The sampling points in our study were set to 2^10^ during KDE, which is close to the median number (1238) of the voxels in 96 regions. The median number represents the general level of the phenomenon relative to other eigenvalues (Kharyutkina et al., 2022). The connectivity were then calculated using the symmetric Kullback-Leibler divergence, which can be mathematically defined as follows:

$$D_{\mathrm{KL}}\left(P,Q\right)=\sum_{i=1}^{n}\left(P\left(i\right)log\frac{P\left(i\right)}{Q\left(i\right)}+Q\left(i\right)log\frac{Q\left(i\right)}{P\left(i\right)}\right)$$

Here, and *n* represents the number of sample points during KDE, and *P* and *Q* represent the probability distribution functions of voxel intensities in a pair of ROIs. Then, the Kullback-Leibler (KLS) similarity score, which represents synaptic density connectivity strength between pairwise ROIs, was normalized within a range of 0 to 1 as follows:

$$KLS\left(P,Q\right)=e^{-D_{\mathrm{KL}}\left(P,Q\right)}$$

Finally, a 96 × 96 undirected weighted matrix representing the pairwise synaptic density connectivity was constructed for each participant (without thresholding). To investigate the impact of key parameters on constructing the individual synaptic density network, we employed an additional range of sampling points during KDE (2^8^–2^11^) for validation purposes. Next, using scripts from the Brain Connectivity Toolbox (Rubinov and Sporns, 2010), we computed nodal degree, which captured the number of significant edges attached to a node. Recognizing the methodological complexity associated with connectivity sparsity threshold selection in network science, we systematically evaluated thresholding effects on synaptic density connectome construction by implementing a comprehensive range of connection densities (50–100 % in 10 % increments) for validation purposes.

### 5.1 Seed-based causal structural covariance network

Additionally, since our connectivity matrices were obtained from the PET imaging of synaptic density, reductions in connectivity alteration could theoretically be related to widespread synaptic perturbations (e.g., synapse loss) in the aforementioned vulnerable networks, we investigated the local synaptic density in regions that showed significant network alterations. Given that ALS is a progressive disease, we then considered whether there was a likely order of synaptic loss in these brain regions. Although the King’s stage simplifies phenotypic heterogeneity in ALS, it is the most straightforward and widely used approach to distinguish the initial, intermediate, and final stages of ALS (Canosa et al., 2021), and it represents, to some extent, the longitudinal progression of the disease (Trojsi et al., 2016). Similar to previous study (Guo et al., 2020, Li et al., 2021), the ALS participants representing the entire disease trajectory were sequenced from low to high according to the King’s stage. Subsequently, the right medulla oblongata with the highest nodal degree was selected as the seed to construct the seed-based cSCN for ALS. At the voxel level, the signed-path coefficient Granger causality analysis (GCA) disposed with an fMRI toolbox (REST; http://www.restfmri.net) was applied to estimate the most probable sequence in the aforementioned brain regions throughout the natural history of ALS. We only included the GC values from X to Y in this research due to the main objective of the study was to investigate the probable sequential patterns of synaptic loss in the medulla oblongata and other synaptic altered region in patients with ALS. The original GC map was z-score-transformed to achieve statistical significance.

### 5.2 ROI-to-ROI causal structural covariance network

Signed-path coefficient GCA was also performed to conduct an ROI-to-ROI causal network on the sequenced morphometric data to further determine the existence of a causal link or causal loop among the specific subcortical and cortical regions. The threshold was set at a GC value > 0.82, which corresponds to voxel wise- cSCN results after False discovery rate (FDR) correction at a threshold of *p* < 0.01. In addition, we calculated the binary in- and out-degrees, which was defined by the total number of inflow/outflow connections to a node region. A region with high out-degree is more likely to be a Granger causal source and a region with a high in-degree is a Granger causal target.

### 5.3 Statistical analysis

To evaluate group differences, we used a two-sample *t*-test for age, a chi-square independence test for gender and a Mann-Whitney test for years of education, depending on whether the data were normally distributed. We also conducted two-sample t-tests to assess group differences in the synaptic density connectome and the local synaptic density in regions. In particular, we performed *z*-tests on the medulla oblongata-associated causal synaptic network. FDR correction was applied to correct for multiple comparisons.

## 6 Results

### 6.1 Demographic and clinical features of subjects

Demographic and clinical information of the 21 ALS patients and 25 HCs with [^18^F]SynVest-1 PET scans are shown in Table 1. The groups did not differ significantly in age (ALS: 52.48 ± 10.98, HC: 51.36 ± 8.47, *p* = 0.699), sex (ALS: 13 males, HC: 13 males, *p* = 0.500) and education (ALS: 8.95 ± 4.28, HC: 9.92 ± 3.00, *p* = 0.111). The age of onset of the ALS patients was 51.05 ± 10.87  years, the time from onset to PET was 19.10 ± 15.12  months, the King’s stage score was 2.38 ± 0.80, and the ALSFRS-R score was 36.33 ± 5.80. In addition, sixteen patients with ALS completed the ECAS, and the ECAS score was 77.81 ± 18.80. The other five patients with ALS failed to complete the ECAS due to severe bulbar and hand dysfunction.

**Table 1:** Clinical and demographic characteristics of participants.

Demographics	ALS (n = 21)	HC (n = 25)	ALS vs HC
	Mean ± SD	Mean ± SD	*p*-value
Age (years)	52.48 ± 10.98	51.36 ± 8.47	0.699^a^
Gender (male/female)	13 / 8	13 / 12	0.500^b^
Education (years)	8.95 ± 4.28	9.92 ± 3.00	0.111^c^
King’s stage	2.38 ± 0.80 (stage I: 2, stage IIa: 2, stage IIb: 9, stage III: 6, stage IVa: 1, stage IVb: 1)	/	/
Onset age (years)	51.05 ± 10.87	/	/
Duration (month)	19.10 ± 15.12	/	/
ALSFRS	36.33 ± 5.80	/	/
ECAS#	77.81 ± 18.80	/	/

Abbreviations: Values are presented as mean ± standard deviation. ALS, amyotrophic lateral sclerosis; HC, healthy controls; ALSFRS, amyotrophic lateral sclerosis functional rating scale; ECAS, Edinburgh Cognitive and Behavioral ALS Screen.

a Two − sample *t* test.

b χ2 test.

c Nonparametric Mann-Whitney test.

# Partial score missed (ECAS n = 16).

### 6.2 ALS-related network changes in the synaptic density connectome

**Fig. 1:**
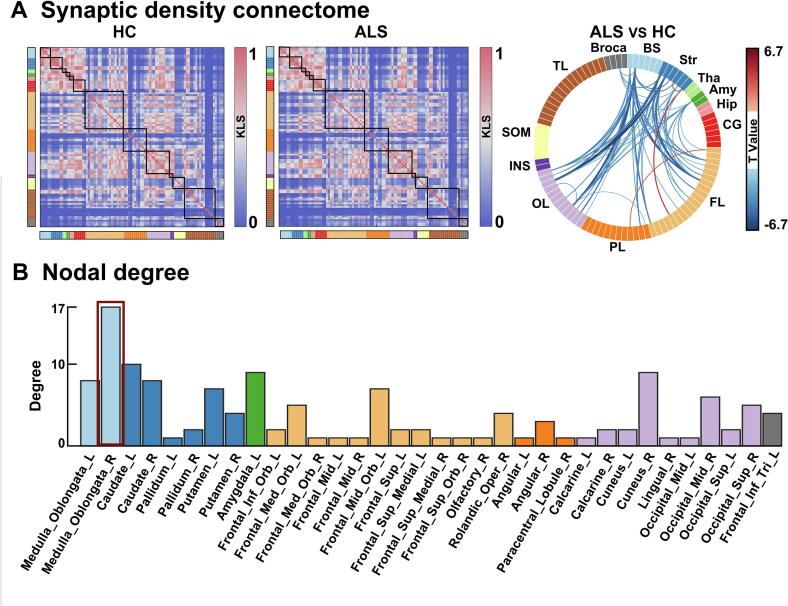
**Synaptic density network dysfunction in ALS.** (a) The synaptic density connectome in the HC group (left) and the ALS group (middle), and the case-control differences (right). The chord diagram was obtained using two-sample t-tests with *p* < 0.01 adjusted for FDR correction. The size of the edges reflects the *t* values for integration associated with significant altered connectivity in ALS versus controls. (b) The bar diagram displays the degree distribution in the chord diagram after excluding zero. HC, healthy controls; ALS, amyotrophic lateral sclerosis; BS, brainstem; Str, striatum; Tha, thalamus; Amy, amygdala; Hip, hippocampus; CG, cingulate cortex; FL, frontal lobe; PL, parietal lobe; OL, occipital lobe; INS, insula; SOM, sensorimotor area; TL, temporal lobe; Broca, broca's area.

We showed that the KLS strength varied widely across brain regions, but was strongest in highly structurally connected visual and subcortical areas (Fig. 1a). Compared to HCs, patients with ALS displayed significantly decreased synaptic density connectome between the striatum and the frontal lobe, occipital lobe, as well as between the bilateral medulla oblongata and subcortical regions (striatum and amygdala), occipital lobe, frontal gyrus, and increased synaptic density connectome between frontal gyrus and parietal lobe (Fig. 1a, *p* < 0.01, FDR corrected). In an exploratory analysis, no significant differences in synaptic density connectivity were found between spinal‐ and bulbar‐onset ALS after FDR correction (all *p* > 0.05). The non-significant results may reflect either insufficient statistical power due to the limited sample size of the bulbar-onset subgroup (n = 6), precluding meaningful between-group comparisons, or alternatively, may suggest reflect a genuine absence of intergroup differences between the two subgroups. Subsequently, we showed the degree distribution of all regions, in which the right medulla oblongata may serve as a hub region, ranking 1th in terms of nodal degree (degree = 17) (Fig. 1b). We further compared the case-control differences at different sampling points (2^8^–2^11^) during KDE and at all connection sparsity, result showed almost replicable alteration patterns in ALS (Supplementary Fig. 1, Supplementary Fig. 2).

### 6.3 Voxel-wise cSCN show a causal effect associated medulla oblongata

**Fig. 2:**
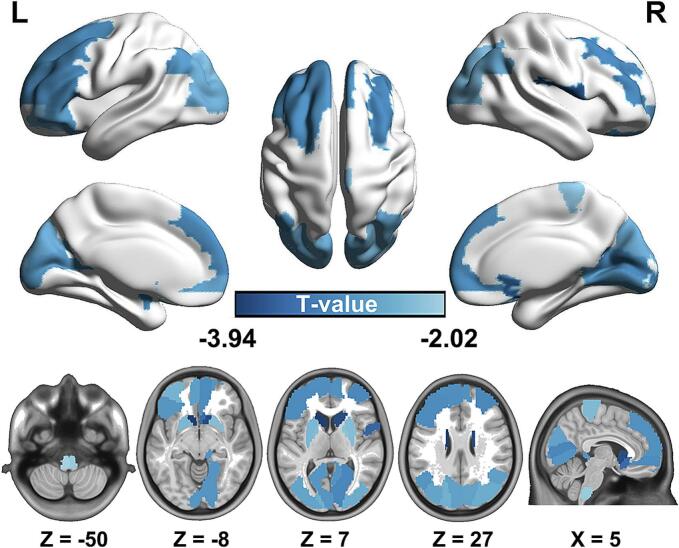
**Synaptic loss in brain regions associated with altered synaptic density connectivity networks in ALS**. Compared with the HCs, patients with ALS displayed significantly reduced synaptic density in bilateral medulla oblongata, striatum, frontal lobe, parietal lobe and occipital lobe. Data were analyzed by using two-sample *t*-tests with *p* < 0.05 adjusted for FDR correction. L, left; R, right.

**Fig. 3:**
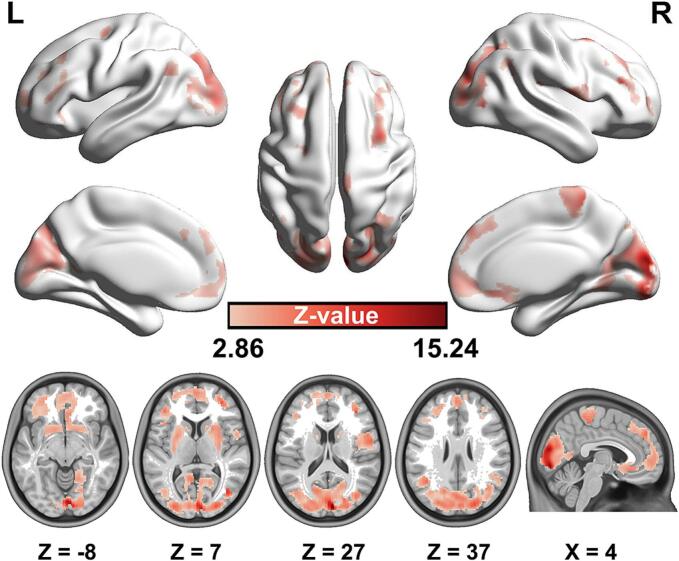
**Voxel-based causal synaptic covariance network analysis in ALS.** Synaptic loss in the right medulla oblongata had causal effects on the bilateral striatum, frontal lobe, parietal lobe as well as occipital lobe. All brain regions were FDR corrected by using *p* < 0.01. Warm color indicates positive Granger causality values. Color bar represents *z-*values transformed from Granger causality values.

Compared to HCs, patients with ALS displayed significantly reduced synaptic density in the 34 brain regions including bilateral medulla oblongata, striatum, frontal lobe, parietal lobe and occipital lobe (Fig. 2, *p* < 0.05, FDR corrected). Subsequently, the cSCN result showed positive GC from the right medulla oblongata to bilateral caudate, putamen, pallidum, frontal lobe, parietal lobe as well as occipital lobe in ALS (*p* < 0.01, FDR corrected, Fig. 3). No negative GC value from the medulla oblongata to other regions was observed.

### 6.4 ROI-wise cSCN reveal interregional causal networks

**Fig. 4:**
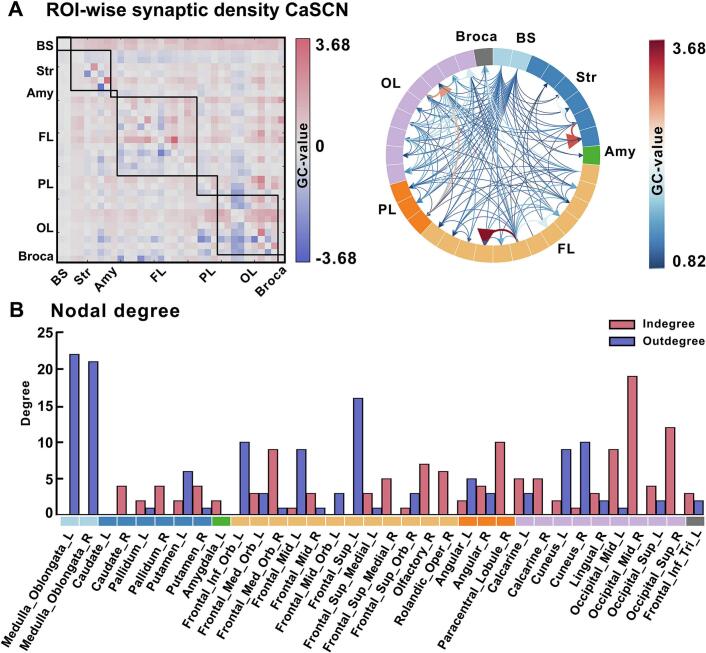
**ROI-wise causal synaptic covariance network analysis in ALS.** Granger causality analysis was performed to construct ROI-wise cSCNs based on King’s stage scores to characterize the interregional synaptic covariance network among ROIs. (a) The heatmap indicated the causal connectivity among ROIs in the ALS group (left) and the chord diagram was obtained using the threshold of GC value > 0.82 (right). The size of the edges reflects strength of the causal connection. (b) Out-in degree of the regions in the ROI-to-ROI causal synaptic covariance network analysis. BS, brainstem; Str, striatum; Amy, amygdala; FL, frontal lobe; PL, parietal lobe; OL, occipital lobe; Broca, broca's area.

**Fig. 5:**
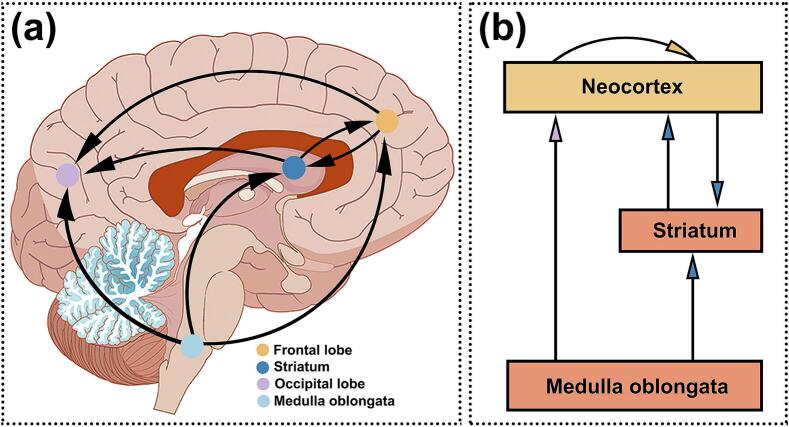
**Probable causal pathways of the synaptic loss in different brain structures associated with medulla oblongata.** (a) Schematic diagram on the sagittal template showing the location of the medulla − striatum − neocortex pathway and the medulla − neocortex pathway. (b) Simplified schematic illustrating these pathways. Directed arrows denote the positive Granger causality between these regions. The striatum includes the caudate, putamen and pallidum. The neocortex includes occipital lobe, frontal gyrus and parietal lobe.

The ROI-to-ROI cSCN result further demonstrated that the medulla oblongata had extensive positive causal influences on the subcortical and cortical networks mentioned above (Fig. 4a). The binary out-in degrees shown in Fig. 4b. No causal effects from other regions to medulla oblongata were found. A schematic causal pathway of the spread of synaptic loss in different brain regions associated with the medulla oblongata was summarized in Fig. 5a. This pathway mainly involves the following two schematic pathways: (i) medulla oblongata − striatum − neocortex; (ii) medulla oblongata – neocortex, which are illustrated in Fig. 5b.

## 7 Discussion

In this study, we characterized the *in vivo* changes in the synaptic density connectome and detected the probable spatial and sequential patterns of synaptic loss at the co-varying network level using [^18^F] SynVesT-1 PET imaging in patients with ALS. Compared to controls, ALS showed an overall decrease in synaptic network connectivity between the medulla oblongata and subcortical regions (striatum and amygdala), frontal lobe, occipital lobe, as well as between the striatum and the frontal lobe, occipital lobe. There was also a significant reduction in synaptic density in the aforementioned brain regions. Furthermore, we demonstrated that the medulla oblongata, which has the highest nodal degree, would drive the late onset synaptic loss in spatially distributed networks including the striatum, frontal lobe, occipital lobe in patients with ALS. To the best of our knowledge, we provide the first known *in vivo* evidence of a loss of synaptic network connectivity and a predictable trajectory of synaptic loss in human ALS patients. Our study may help to understand the mechanism of synaptic network degeneration in the progression of ALS.

By constructing an individual whole-brain synaptic connectome based on [^18^F]SynVesT-1 PET images and applying nodal degree analyses, we have further identified widespread network alterations in synaptic connectivity in ALS patients. The observed aberrant connections were predominantly found in the basal ganglia-neocortical and brainstem-neocortical networks. Prior neuroimaging evidence from diffusion MRI study has shown decreased structural connectivity between the caudate head and the dorsomedial frontal cortex, the lateral orbitofrontal cortex was found in ALS patients (Masuda et al., 2016), which were considered central to cognitive dysfunction in ALS (Machts et al., 2015). Notably, although a wide range of brain structures were involved in synaptic density similarity network changes in ALS patients, we found that these effects were most pronounced in the medulla oblongata, which had the highest nodal degree. Altered structural and functional connectivity in the cortico-brainstem circuit in ALS patients was identified from a network perspective (Schmidt et al., 2014, Tahedl et al., 2023). By identifying complex synaptic network, our results extended the previous study on network-level alterations in brain connectivity derived from magnetic resonance (MR) data. ALS should be considered as a result of a system degeneration due to network failure instead of focusing on individual neuronal populations (Turner et al., 2013). Overall, we provides molecular imaging evidence for the network theory of ALS showing damaged connectivity in a spatially distributed network. In addition, since our synaptic network connectivity were derived from the PET imaging of synaptic density, reductions in strength could be related to widespread synaptic perturbations (e.g., synapse loss) in the fragile hubs described above. The results were largely consistent with findings from animal models of disease and ALS patients, which have shown synaptic loss in the prefrontal cortex, brainstem, striatum, postcentral neocortex, and temporal lobe (Brettschneider et al., 2013, Henstridge et al., 2018) (Liu et al., 2016). These regions have typically been implicated in ALS pathogenesis (Henstridge et al., 2018, Susan et al., 2014, Tang et al., 2022a, Wang et al., 2022).

Longitudinal studies have reported progressive regional degeneration in the key brain regions, including the brainstem (medulla oblongata), insular cortex and bilateral caudate (Bede et al., 2019, Ishaque et al., 2021), but the causal neurobiological alterations underlying ALS pathogenesis remain incompletely characterized. To investigate the most probable sequence of synaptic loss in these abnormally connected hubs, we offered a novel perspective on the network degeneration in ALS by proposing a strategy of cSCN on cross-sectional PET data. The medulla oblongata has the highest nodal degree, suggesting its potential to expand our understanding of the origin, spread, and early clinical diagnosis of ALS. Therefore, we investigated the causal patterns of synaptic loss associated with the medulla oblongata. Notably, we showed that synaptic loss in the medulla oblongata has significant positive causal influence on the subcortical and cortical networks. Our findings broadly support the 'bottom-up-theory' that the altered processing of sensory inputs at the brainstem level in ALS (Hübers et al., 2016). The reticular formation of the brainstem is an extended ill-defined network of interconnected neurons comprising the complete spectrum of neurons, from small- to giant-sized, and is associated with rapid disease progression (Grinberg et al., 2011). The specific reticular nuclei produce aminergic transmitters that are thought to project diffusely into the brain through“ volume transmission” and may affect a larger number of receptors (Mountcastle, 1998). This influence may be mediated directly or indirectly through axonal projections from the medulla oblongata to other regions, which could serve as pre-existing pathways to canalize dissemination of synaptic loss in ALS.

Probing interregional relationships of the probable sequence within pre-existing brain networks may provide a deeper understanding of the natural history and behavioral consequences in neurodegenerative diseases, in which pathology targets and spreads along particular networks. The ROI-based cSCN further revealed that the brainstem, as the hub of the directional network, had extensive positive causal effects on other regions, particularly the basal ganglia (bilateral pallidum, left amygdala), frontal gyrus, bilateral angular gyrus and occipital lobe. We observed two main distal pathways that spreading from the medulla oblongata (i) directly to the frontal gyrus and occipital lobe; (ii) indirectly through the striatum to the frontal gyrus and occipital lobe. The current results fit well with the previously proposed phosphorylated 43-kDa transactive response DNA-binding protein (pTDP-43) pathological staging scheme, with original lesions in the medulla oblongata, and progression to the prefrontal neocortex (middle frontal gyrus, gyrus rectus and orbital gyri), striatum during the subsequent stages (Brettschneider et al., 2013). The underlying mechanisms of the pathological process are far from clear, and we discuss some hypotheses below. First, ALS may spread contiguously or non-contiguously following a ‘domino-prion-like’ propagation between neighboring neurons in a cell autonomous or non-cell autonomous manner, mutant SOD1 and normal TDP-43 can undergo a seeded aggregation similar to prions(Münch et al., 2011), and initiate the disease cascade that may lead to synaptic dysfunction and cell death. Second, upper motor neurons in the cerebral cortex make direct (monosynaptic) or indirect (via interneurons) connections with lower motor neurons in the brainstem and spinal cord (Verma et al., 2022), several cells play a role in synaptic communication thoughout the motor neuro network system including interneurons, astrocytes, microglia, schwann and skeletal muscle cells. These interconnected cells with long projections require large amounts of energy to maintain their homeostasis, and are more vulnerable to mitochondrial dysfunction and axonal dysfunction (Yamanaka and Komine, 2018). Third, it is thought that the remote propagation relies on remote transfer of a toxic molecule through the blood or CSF (Kanouchi et al., 2012). Overall, pathology could disrupt the integrity of the networks in which they participate in, leading to alterations in spatially remote, functionally related regions via contiguous type propagation, *trans*-synaptic type propagation, non-synaptic type propagation.

Some limitations should be noted. Firstly, we acknowledge that the timing of PET scans relative to treatment initiation may influence synaptic density measures, as suggested by longitudinal MRI study (Distaso et al., 2021). By reporting the diagnosis-to-imaging interval, we aim to facilitate cross-study comparisons and inform the design of future longitudinal biomarker investigations. Secondly, given the uneven proportion of ALS patients in the King’s stages (Gebrehiwet et al., 2023, Roche et al., 2012) and the challenges of repeated neuroimaging in patients with bulbar and respiratory compromise (Turner and Kiernan, 2012), the sample size within specific King's stages and the bulbar-onset subgroup remains limited. While sufficient to detect significant synaptic reductions, larger longitudinal studies are needed to confirm and refine the trajectory of brain modifications during disease progression. Thirdly, SV2A PET provides a quantitative readout of synaptic density but does not directly assay synaptic efficacy or neurotransmission. Future studies integrating SV2A imaging with functional modalities such as [^18^F]FDG PET, magnetic resonance spectroscopy, or electrophysiological assessments will be valuable for disentangling the relationship between structural synaptic loss and functional impairment in ALS. Finally, the major caveat in interpreting GCA results based on cross-sectional data involves the indirect nature of this method, and itself alone does not constitute proof of the equivalence to pathological causal interaction. Further work should incorporate serial PET scans, stratification by clinical subtype, and cross-disease comparisons to establish robust biomarkers of ALS progression.

## 8 Conclusion

Collectively, our study shows the *in vivo* spatial patterns of synaptic network alterations and the probable sequences of synaptic loss in patients with ALS. Synaptic network alterations are primarily distributed in the medulla oblongata, striatum and neocortical networks. Notably, synaptic loss in these networks occurs in a specific manner and propagates in a consistent order, targeting the medulla oblongata-striatum-neocortical network in ALS patients. These findings provide a new perspective on the natural history of neurodegeneration in ALS and may help to slow or even reverse disease-related neurological changes in the target network.

**Funding**

This research was supported by the National Science and Technology Innovation 2030 Major Program (Nos.2022ZD0208903), the National Natural Science Foundation of China (Nos. 62036003, 62333003, 82372085, 82272045, 81801740), the Sichuan Science and Technology Foundation (No. 2023NSFSC0644), the Science and Technology Innovation Program of Hunan Province (2021RC4056), the Key Program of Ministry of Industry and Information Technology of China (CEIEC-2022-ZM02-0219), the Hunan Provincial Science Fund for Distinguished Young Scholars (2024JJ2094), the Clinical Research Foundation of the National Clinical Research Center for Geriatric Diseases (XIANGYA) (No. 2023LNJJ16), the China Postdoctoral Science Foundation (No. 2022M723561), and the National Key Clinical Specialty Scientific Research Project (No. Z2023004).

## CRediT authorship contribution statement

**Ting Zou:** Writing – original draft, Visualization, Validation, Methodology, Formal analysis, Conceptualization. **Manliu Hou:** Writing – review & editing, Visualization, Validation, Investigation, Data curation. **Honghao Han:** Writing – review & editing, Visualization, Validation, Methodology, Investigation. **Xuyang Wang:** Writing – review & editing, Visualization, Validation, Methodology, Conceptualization. **Huafu Chen:** Writing – review & editing, Supervision, Funding acquisition, Conceptualization. **Yongxiang Tang:** Writing – review & editing, Supervision, Funding acquisition, Data curation. **Rong Li:** Writing – review & editing, Supervision, Funding acquisition, Conceptualization. **Shuo Hu:** Writing – review & editing, Supervision, Funding acquisition, Data curation.

## Declaration of competing interest

The authors declare that they have no known competing financial interests or personal relationships that could have appeared to influence the work reported in this paper.

## Data Availability

The data that support the findings of this study is available from the corresponding author upon reasonable request. The codes for constructing the synaptic density connectome using KLSE can be found at (https://github.com/RongLi1120/KLSE).

## References

[b0005] Abrahams S., Newton J., Niven E., Foley J., Bak T.H. (2014). Screening for cognition and behaviour changes in ALS. Amyotrophic Lateral Sclerosis and Frontotemporal Degeneration.

[b0010] Basaia S., Agosta F., Cividini C., Trojsi F., Riva N., Spinelli E.G., Moglia C., Femiano C., Castelnovo V., Canu E. (2020). Structural and functional brain connectome in motor neuron diseases: a multicenter MRI study. Neurology.

[b0015] Bede P., Chipika R.H., Finegan E., Shing S.L.H., Doherty M.A., Hengeveld J.C., Vajda A., Hutchinson S., Donaghy C., McLaughlin R.L. (2019). Brainstem pathology in amyotrophic lateral sclerosis and primary lateral sclerosis: a longitudinal neuroimaging study. NeuroImage: Clinical.

[b0020] Braak H., Brettschneider J., Ludolph A.C., Lee V.M., Trojanowski J.Q., Tredici K.D. (2013). Amyotrophic lateral sclerosis—a model of corticofugal axonal spread. Nat. Rev. Neurol..

[b0025] Brettschneider J., Del Tredici K., Toledo J.B., Robinson J.L., Irwin D.J., Grossman M., Suh E., Van Deerlin V.M., Wood E.M., Baek Y. (2013). Stages of pTDP‐43 pathology in amyotrophic lateral sclerosis. Ann. Neurol..

[b0030] Canosa A., Calvo A., Moglia C., Manera U., Vasta R., Di Pede F., Cabras S., Nardo D., Arena V., Grassano M. (2021). Brain metabolic changes across king’s stages in amyotrophic lateral sclerosis: a 18 F-2-fluoro-2-deoxy-D-glucose-positron emission tomography study. Eur. J. Nucl. Med. Mol. Imaging.

[b0035] De Lorenzo F., Lüningschrör P., Nam J., Beckett L., Pilotto F., Galli E., Lindholm P., Rüdt von Collenberg C., Mungwa S.T., Jablonka S. (2023). CDNF rescues motor neurons in models of amyotrophic lateral sclerosis by targeting endoplasmic reticulum stress. Brain.

[b0040] Distaso E., Milella G., Mezzapesa D.M., Introna A., D’Errico E., Fraddosio A., Zoccolella S., Dicuonzo F., Simone I.L. (2021). Magnetic resonance metrics to evaluate the effect of therapy in amyotrophic lateral sclerosis: the experience with edaravone. J. Neurol..

[b0045] Feldman E.L., Goutman S.A., Petri S., Mazzini L., Savelieff M.G., Shaw P.J., Sobue G. (2022). Amyotrophic lateral sclerosis. Lancet.

[b0050] Gebrehiwet P., Meng L., Rudnicki S.A., Sarocco P., Wei J., Wolff A.A., Chiò A., Andrews J.A., Genge A., Jackson C.E. (2023). MiToS and king’s staging as clinical outcome measures in ALS: a retrospective analysis of the FORTITUDE-ALS trial. Amyotrophic Lateral Sclerosis and Frontotemporal Degeneration.

[b0055] Geevasinga N., Menon P., Van Den Bos M., Gomes L., Forster S., Kiernan M., Vucic S. (2017).

[b0060] Grinberg L.T., Rueb U., Heinsen H. (2011). Brainstem: neglected locus in neurodegenerative diseases. Front. Neurol..

[b0065] Guo J., Chen H., Biswal B.B., Guo X., Zhang H., Dai L., Zhang Y., Li L., Fan Y., Han S. (2020). Gray matter atrophy patterns within the cerebellum-neostriatum-cortical network in SCA3. Neurology.

[b0070] Hübers A., Kassubek J., Grön G., Gorges M., Aho-Oezhan H., Keller J., Horn H., Neugebauer H., Uttner I., Lulé D. (2016). Pathological laughing and crying in amyotrophic lateral sclerosis is related to frontal cortex function. J. Neurol..

[b0075] Henstridge C.M., Pickett E., Spires-Jones T.L. (2016). Synaptic pathology: a shared mechanism in neurological disease. Ageing Res. Rev..

[b0080] Henstridge C.M., Sideris D.I., Carroll E., Rotariu S., Salomonsson S., Tzioras M., McKenzie C.-A., Smith C., von Arnim C.A., Ludolph A.C. (2018). Synapse loss in the prefrontal cortex is associated with cognitive decline in amyotrophic lateral sclerosis. Acta Neuropathol..

[b0085] Hoye M.L., Regan M.R., Jensen L.A., Lake A.M., Reddy L.V., Vidensky S., Richard J.-P., Maragakis N.J., Rothstein J.D., Dougherty J.D. (2018). Motor neuron-derived microRNAs cause astrocyte dysfunction in amyotrophic lateral sclerosis. Brain.

[b0090] Ishaque A., Ta D., Khan M., Zinman L., Korngut L., Genge A., Dionne A., Briemberg H., Luk C., Yang Y.H., Beaulieu C., Emery D., Eurich D.T., Frayne R., Graham S., Wilman A., Dupré N., Kalra S. (2021). Distinct patterns of progressive gray and white matter degeneration in amyotrophic lateral sclerosis. Hum. Brain Mapp..

[b0095] Kanouchi T., Ohkubo T., Yokota T. (2012). Can regional spreading of amyotrophic lateral sclerosis motor symptoms be explained by prion-like propagation?. J. Neurol. Neurosurg. Psychiatry.

[b0100] Kharyutkina E., Loginov S., Martynova Y., Sudakov I. (2022). Time series analysis of atmospheric precipitation characteristics in Western Siberia for 1979–2018 across different datasets. Atmos..

[b0105] Lépine S., Castellanos-Montiel M.J., Durcan T.M. (2022). TDP-43 dysregulation and neuromuscular junction disruption in amyotrophic lateral sclerosis. Translational Neurodegeneration.

[b0110] Li R., Xiao L., Han H., Long H., Liao W., Yang Z., Zhu H., Wang X., Zou T., Huang Y. (2025). Transcriptionally downregulated GABAergic genes associated with synaptic density network dysfunction in temporal lobe epilepsy. Eur. J. Nucl. Med. Mol. Imaging.

[b0115] Li R., Zou T., Wang X., Wang H., Hu X., Xie F., Meng L., Chen H. (2021). Basal ganglia atrophy–associated causal structural network degeneration in Parkinson's disease. Hum. Brain Mapp..

[b0120] Li S., Cai Z., Wu X., Holden D., Pracitto R., Kapinos M., Gao H., Labaree D., Nabulsi N., Carson R.E. (2018). Synthesis and in vivo evaluation of a novel PET radiotracer for imaging of synaptic vesicle glycoprotein 2A (SV2A) in nonhuman primates. ACS Chem. Nerosci..

[b0125] Liu Y., Pattamatta A., Zu T., Reid T., Bardhi O., Borchelt D.R., Yachnis A.T., Ranum L.P. (2016). C9orf72 BAC mouse model with motor deficits and neurodegenerative features of ALS/FTD. Neuron.

[b0130] Ludolph A., Drory V., Hardiman O., Nakano I., Ravits J., Robberecht W., Shefner J., ALS/MND, W.R.G.O. (2015). A revision of the El Escorial criteria-2015. Amyotrophic Lateral Sclerosis and Frontotemporal Degeneration.

[b0135] Münch C., O’Brien J., Bertolotti A. (2011). Prion-like propagation of mutant superoxide dismutase-1 misfolding in neuronal cells. Proc. Natl. Acad. Sci..

[b0140] Machts J., Loewe K., Kaufmann J., Jakubiczka S., Abdulla S., Petri S., Dengler R., Heinze H.-J., Vielhaber S., Schoenfeld M.A., Bede P. (2015). Basal ganglia pathology in ALS is associated with neuropsychological deficits. Neurology.

[b0145] Masuda M., Senda J., Watanabe H., Epifanio B., Tanaka Y., Imai K., Riku Y., Li Y., Nakamura R., Ito M., Ishigaki S., Atsuta N., Koike H., Katsuno M., Hattori N., Naganawa S., Sobue G. (2016). Involvement of the caudate nucleus head and its networks in sporadic amyotrophic lateral sclerosis-frontotemporal dementia continuum. Amyotrophic Lateral Sclerosis and Frontotemporal Degeneration.

[b0150] Mertens N., Sunaert S., Van Laere K., Koole M. (2022). The effect of aging on brain glucose metabolic connectivity revealed by [18F] FDG PET-MR and individual brain networks. Front. Aging Neurosci..

[b0155] Mountcastle V.B. (1998).

[b0160] Naganawa M., Li S., Nabulsi N., Henry S., Zheng M.-Q., Pracitto R., Cai Z., Gao H., Kapinos M., Labaree D. (2021). First-in-human evaluation of 18F-SynVesT-1, a radioligand for PET imaging of synaptic vesicle glycoprotein 2A. J. Nucl. Med..

[b0165] Roche J.C., Rojas-Garcia R., Scott K.M., Scotton W., Ellis C.E., Burman R., Wijesekera L., Turner M.R., Leigh P.N., Shaw C.E. (2012). A proposed staging system for amyotrophic lateral sclerosis. Brain.

[b0170] Rubinov M., Sporns O. (2010). Complex network measures of brain connectivity: uses and interpretations. Neuroimage.

[b0175] Schmidt R., Verstraete E., de Reus M.A., Veldink J.H., van den Berg L.H., van den Heuvel M.P. (2014). Correlation between structural and functional connectivity impairment in amyotrophic lateral sclerosis. Hum. Brain Mapp..

[b0180] Shima K., Matsunari I., Samuraki M., Chen W.P., Yamada M. (2012). Posterior cingulate atrophy and metabolic decline in early stage Alzheimer's disease. Neurobiol. Aging.

[b0185] Susan B., Peter B., Daniel G., Bradley M., Elamin R. (2014). Basal ganglia involvement in amyotrophic lateral sclerosis. Neurol. Official J. Am. Acad. Neurol..

[b0190] Tahedl M., Tan E.L., Chipika R.H., Hengeveld J.C., Vajda A., Doherty M.A., McLaughlin R.L., Siah W.F., Hardiman O., Bede P. (2023). Brainstem–cortex disconnection in amyotrophic lateral sclerosis: bulbar impairment, genotype associations, asymptomatic changes and biomarker opportunities. J. Neurol..

[b0195] Tang Y., Liu P., Li W., Liu Z., Zhou M., Li J., Yuan Y., Fang L., Wang M., Shen L. (2022). Detection of changes in synaptic density in amyotrophic lateral sclerosis patients using 18F‐SynVesT‐1 positron emission tomography. Eur. J. Neurol..

[b0200] Tang Y., Yu J., Zhou M., Chen C., Hu S. (2022). 18F-SynVesT-1 PET in focal cortical dysplasia type II with thickening cortex. Clin. Nucl. Med..

[b0205] Tang Y., Yu J., Zhou M., Li J., Long T., Li Y., Feng L., Chen D., Yang Z., Huang Y. (2022). Cortical abnormalities of synaptic vesicle protein 2A in focal cortical dysplasia type II identified in vivo with 18F-SynVesT-1 positron emission tomography imaging. Eur. J. Nucl. Med. Mol. Imaging.

[b0210] Trojsi F., Santangelo G., Caiazzo G., Siciliano M., Ferrantino T., Piccirillo G., Femiano C., Cristillo V., Monsurrò M.R., Esposito F. (2016). Neuropsychological assessment in different king's clinical stages of amyotrophic lateral sclerosis. Amyotrophic Lateral Sclerosis and Frontotemporal Degeneration.

[b0215] Turner M.R., Hardiman O., Benatar M., Brooks B.R., Chio A., Carvalho M.D., Ince P.G., Lin C., Miller R.G., Mitsumoto H. (2013).

[b0220] Turner M.R., Kiernan M.C. (2012). Does interneuronal dysfunction contribute to neurodegeneration in amyotrophic lateral sclerosis?. Amyotroph. Lateral Scler..

[b0225] Verma S., Khurana S., Vats A., Sahu B., Ganguly N.K., Chakraborti P., Gourie-Devi M., Taneja V. (2022). Neuromuscular junction dysfunction in amyotrophic lateral sclerosis. Mol. Neurobiol..

[b0230] Wang L., Zhou C., Cheng W., Rolls E.T., Huang P., Ma N., Liu Y., Zhang Y., Guan X., Guo T. (2022). Dopamine depletion and subcortical dysfunction disrupt cortical synchronization and metastability affecting cognitive function in Parkinson's disease. Hum. Brain Mapp..

[b0235] Wang M., Jiang J., Yan Z., Alberts I., Ge J., Zhang H., Zuo C., Yu J., Rominger A., Shi K. (2020). Individual brain metabolic connectome indicator based on kullback-leibler divergence Similarity estimation predicts progression from mild cognitive impairment to Alzheimer’s dementia. Eur. J. Nucl. Med. Mol. Imaging.

[b0240] Yamanaka K., Komine O. (2018). The multi-dimensional roles of astrocytes in ALS. Neurosci. Res..

[b0245] Ye S., Ji Y., Li C., He J., Liu X., Fan D. (2016). The Edinburgh cognitive and behavioural ALS screen in a Chinese amyotrophic lateral sclerosis population. PLoS One.

