# Supplementary material for: Medulla oblongata dominated synaptic density network degeneration in amyotrophic lateral sclerosis
Source: Neuroimage Clin. 2025 Jun 18;47:103814. doi: 10.1016/j.nicl.2025.103814 (PMC12216726; doi:10.1016/j.nicl.2025.103814)
Supplement: Supplementary Data 1 [file mmc1.docx]

**Medulla oblongata dominated synaptic density network degeneration in** **amyotrophic lateral sclerosis**

Running title: Medulla oblongata-associated cSCN in ALS

Ting Zou, PhD ^1,2†^, Manliu Hou, MS ^3,4†^, Honghao Han, PhD ^1,2^, Xuyang Wang, PhD ^1,2^, Huafu Chen, PhD ^1,2^*, Yongxiang Tang, MD, PhD ^3,4,5^*, Rong Li, PhD ^1,2^*, Shuo Hu, MD, PhD ^3,4,6^*

^1^ The Clinical Hospital of Chengdu Brain Science Institute, School of Life Science and Technology, University of Electronic Science and Technology of China, Chengdu, 610054, P.R. China.

^2^ MOE Key Laboratory for Neuroinformation, High-Field Magnetic Resonance Brain Imaging Key Laboratory of Sichuan Province, University of Electronic Science and Technology of China, Chengdu, 610054, P.R. China.

^3^ Department of Nuclear Medicine, Xiangya Hospital, Central South University, Changsha, 410008, P.R. China.

^4^ National Clinical Research Center for Geriatric Diseases, Xiangya Hospital, Central South University, Changsha, 410008, P.R. China.

^5^ Department of Nuclear Medicine, Inselspital, University Hospital Bern, Bern, Switzerland.

^6^ Key Laboratory of Biological Nanotechnology of National Health Commission, Xiangya Hospital, Central South University, Changsha, 410008, P.R. China.

* Corresponding Author: Shuo Hu, email: [hushuo2018@163.com](H:/ALS/ALS_HC/ZOUTING/AAL90_newlabel/初稿/12_Neuroimage/hushuo2018@163.com); Rong Li, email: [rongli1120@gmail.com](mailto:rongli1120@gmail.com); Yongxiang Tang, email: [405035@csu.edu.cn](H:/ALS/ALS_HC/ZOUTING/AAL90_newlabel/初稿/12_Neuroimage/405035@csu.edu.cn); Huafu Chen, email: [chenhf@uestc.edu.cn](H:/ALS/ALS_HC/ZOUTING/AAL90_newlabel/初稿/12_Neuroimage/chenhf@uestc.edu.cn)

† These authors contributed equally to this work

**Supplementary Table**

**Supplementary Table 1** Clinical and demographic characteristics of all ALS patient (N = 21)

| Case | f/m | Age | Education  (year) | Age at onset | Onset | King' stage | ALSFRS | Duration (year) | ECAS | Drug | Time Interval (Treatment to Scan, Day) |
| --- | --- | --- | --- | --- | --- | --- | --- | --- | --- | --- | --- |
| 1 | m | 44 | 9 | 44 | Spinal | 1 | 45 | 4 | 96 | Riluzole + Edaravone | 12 |
| 2 | f | 62 | 6 | 62 | Bulbar | 1 | 40 | 7 | - | Riluzole + Edaravone | 14 |
| 3 | m | 51 | 19 | 51 | Spinal | 2A | 38 | 8 | 116 | Riluzole | 231 |
| 4 | m | 61 | 1 | 61 | Spinal | 2A | 41 | 3 | 77 | Riluzole + Edaravone | 1 |
| 5 | m | 57 | 6 | 55 | Spinal | 2B | 26 | 24 | 54 | Riluzole + Edaravone | 539 |
| 6 | m | 63 | 6 | 58 | Spinal | 2B | 40 | 60 | 72 | Riluzole | 3 |
| 7 | m | 56 | 9 | 55 | Bulbar | 2B | 37 | 12 | 56 | Riluzole | 154 |
| 8 | m | 39 | 9 | 38 | Bulbar | 2B | 37 | 9 | - | Riluzole + Edaravone | 11 |
| 9 | m | 35 | 17 | 33 | Spinal | 2B | 42 | 36 | 93 | Riluzole + Edaravone | 22 |
| 10 | f | 68 | 6 | 64 | Spinal | 2B | 39 | 48 | 87 | Riluzole | 486 |
| 11 | m | 35 | 8 | 33 | Spinal | 2B | 37 | 28 | 70 | Riluzole + Edaravone | 1 |
| 12 | f | 59 | 12 | 58 | Spinal | 2B | 39 | 15 | - | Riluzole + Edaravone | 9 |
| 13 | m | 52 | 9 | 52 | Spinal | 2B | 38 | 4 | 95 | Riluzole + Edaravone | 1 |
| 14 | f | 49 | 5 | 49 | Spinal | 3 | 35 | 6 | 69 | Riluzole | 78 |
| 15 | m | 70 | 9 | 69 | Spinal | 3 | 41 | 12 | 56 | Edaravone | 4 |
| 16 | f | 40 | 9 | 38 | Bulbar | 3 | 38 | 22 | 103 | Riluzole + Edaravone | 550 |
| 17 | f | 66 | 9 | 65 | Bulbar | 3 | 38 | 12 | - | Edaravone | 4 |
| 18 | m | 51 | 17 | 48 | Spinal | 3 | 27 | 25 | 75 | Riluzole + Edaravone | 658 |
| 19 | f | 57 | 9 | 55 | Spinal | 3 | 21 | 21 | - | Riluzole + Edaravone | 7 |
| 20 | m | 51 | 6 | 49 | Bulbar | 4A | 32 | 32 | 71 | Riluzole + Edaravone | 505 |
| 21 | f | 36 | 7 | 35 | Spinal | 4B | 32 | 13 | 55 | Riluzole | 119 |

Abbreviations: case, case number; ALS, amyotrophic lateral sclerosis; f/m. female/male; ALSFRS-R, amyotrophic lateral sclerosis functional rating scale-revised; ECAS, Edinburgh Cognitive and Behavioral ALS Screen.

**Supplementary Figure**

#
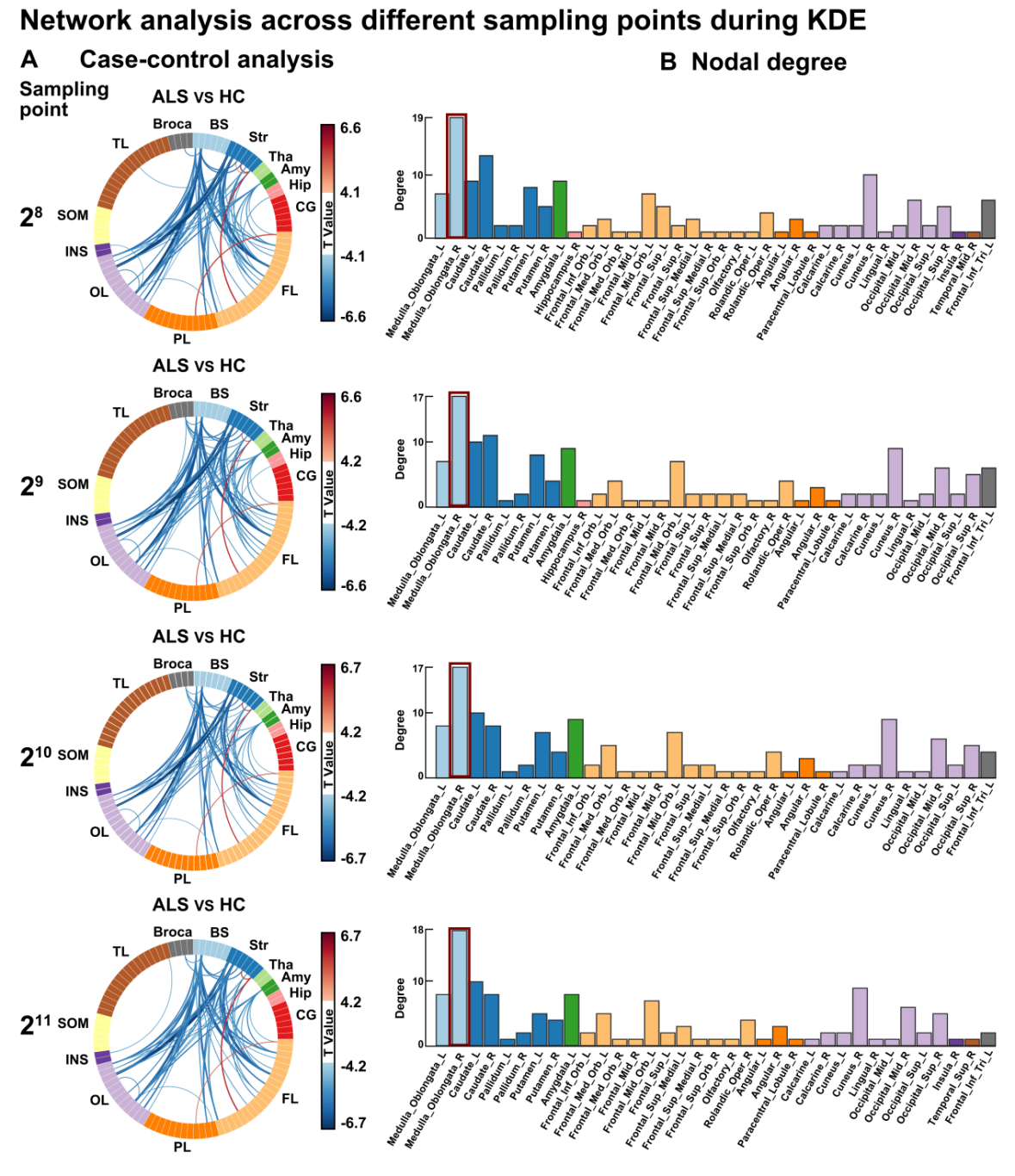


**Supplementary Fig. 1. Replicable case-control network analysis in synaptic density networks across different sampling points during KDE.** (A) Case-control differences in synaptic density networks with an additional range of sampling points during KDE (2^11^-2^8^). All *p*-value were FDR corrected by using *p* < 0.01. (B) Degree distribution of brain regions related to the change of synaptic density network connection at the nodal level with an additional range of sampling points during KDE (2^8^-2^11^). KDE, Kernel Density Estimation. HC, healthy controls; ALS, amyotrophic lateral sclerosis; BS, brainstem; Str, striatum; Tha, thalamus; Amy, amygdala; Hip, hippocampus; CG, cingulate cortex; FL, frontal lobe; PL, parietal lobe; OL, occipital lobe; INS, insula; SOM, sensorimotor area; TL, temporal lobe; Broca, broca's area.


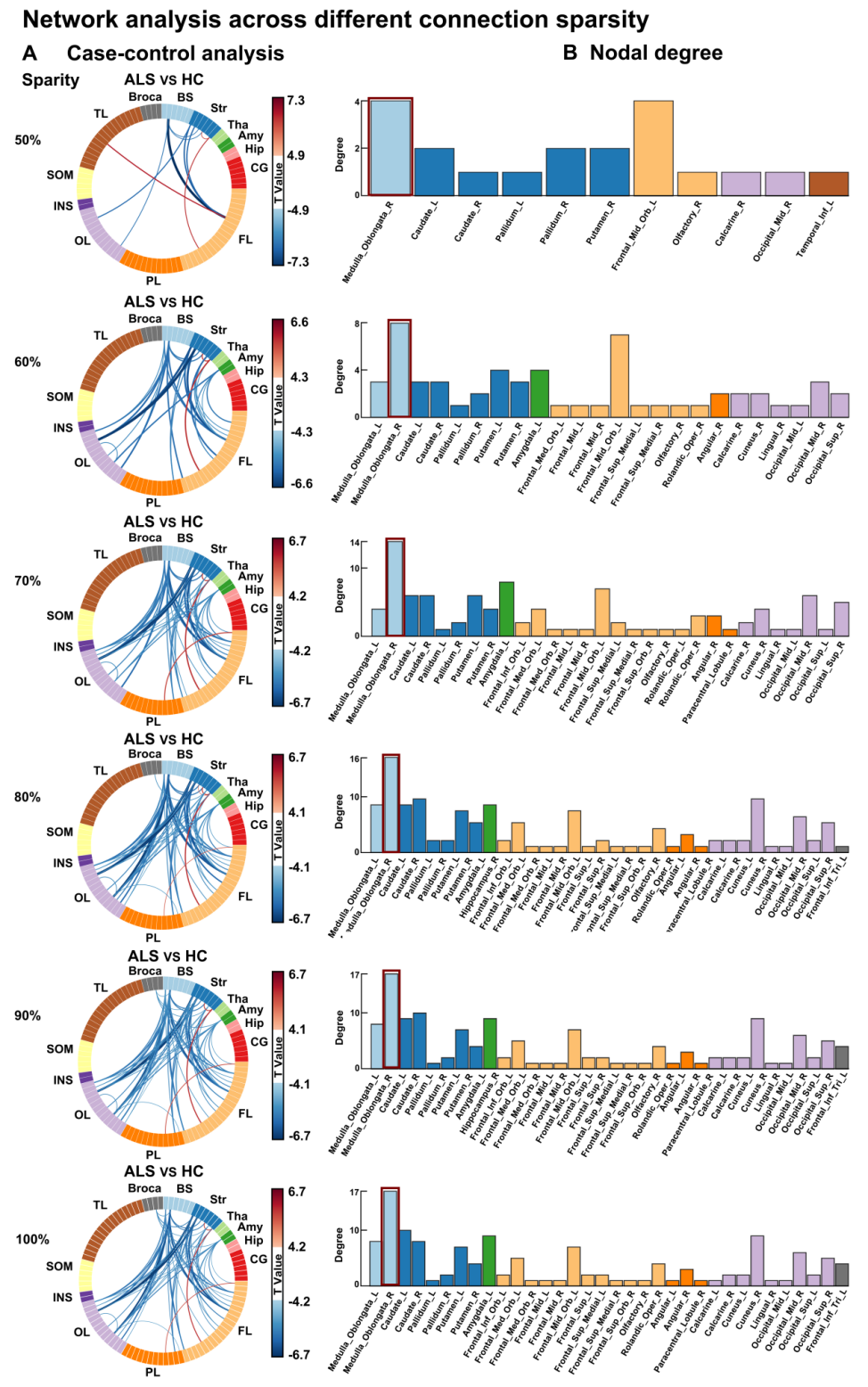


**Supplementary Fig 2. Similar case-control analysis in synaptic density networks across different connection sparsity.** (A) Case-control differences in synaptic density networks with an additional range of connection sparsity (50-100%, in 10% increments). All *p*-value were FDR corrected by using *p* < 0.01. (B) Degree distribution of brain regions related to the changes of synaptic density network connection at the nodal level with an additional range of connection sparsity (50-100%, in 10% increments). HC, healthy controls; ALS, amyotrophic lateral sclerosis; BS, brainstem; Str, striatum; Tha, thalamus; Amy, amygdala; Hip, hippocampus; CG, cingulate cortex; FL, frontal lobe; PL, parietal lobe; OL, occipital lobe; INS, insula; SOM, sensorimotor area; TL, temporal lobe; Broca, broca's area.
